# Smart City Solutions from a Societal Perspective—A Case Study

**DOI:** 10.3390/ijerph20065136

**Published:** 2023-03-14

**Authors:** Agnieszka Szczepańska, Rafał Kaźmierczak, Monika Myszkowska

**Affiliations:** 1 Department of Socio-Economic Geography, Institute of Spatial Management and Geography, Faculty of Geoengineering, University of Warmia and Mazury in Olsztyn, Prawocheńskiego 15, 10-724 Olsztyn, Poland; 2 Department of Land Management and Geographic Information Systems, Institute of Spatial Management and Geography, Faculty of Geoengineering, University of Warmia and Mazury in Olsztyn, Prawocheńskiego 15, 10-724 Olsztyn, Poland; 3 Scientific Circle of Spatial Management, Institute of Spatial Management and Geography, Faculty of Geoengineering, University of Warmia and Mazury in Olsztyn, Prawocheńskiego 15, 10-724 Olsztyn, Poland

**Keywords:** smart city, smart solutions, quality of life, society

## Abstract

More and more cities are evolving towards the smart city concept, which brings about a tangible effect of raised life quality levels. This is confirmed by the satisfaction of inhabitants with the introduced smart solutions. It is their opinion on this issue that is crucial, as they are the main beneficiaries of these measures. This article presents a case study of a medium-sized city, which illustrates the smart city issue from an inhabitants’ perspective. An analysis of objective indices classifies a city as smart, and it is included in European lists of smart cities. It is interesting, however, to see how the inhabitants themselves assess the city in the context of the smart solutions in place. Are they relevant to them? Has the quality of life increased? Are they satisfied with the individual aspects of city functioning? What solutions are they awaiting? What areas require changes? The involvement of inhabitants and the public participation level were also assessed. The questionnaire survey results indicated whether the city can be classified as a smart city of the future and identified the spheres of the city’s activities that need improvement. The main conclusions are that inhabitants have a positive perception of a city’s smart services provided that their implementation results in an enhanced quality of life. At the same time, even though inhabitants are aware of the smart services introduced, they do not fully use them, which may be due to their low attractiveness, a lack of promotion on the part of the city, or a lack of equipment readiness.

## 1. Introduction

Cities are becoming increasingly important centres of economic, cultural, and social development. However, their rapid development without applying appropriate techniques, solutions, and management is characterised by chaotic, disorderly sprawls of buildings, traffic paralysis, and environmental degradation. All of this significantly contributes to lowering the standards of life quality for inhabitants. For this reason, the smart city concept that introduces thoughtful, modern ways of urban management is a necessity in the development of urban areas. The main reason for the development of this concept is the growing interest in the city as a place to live. Due to the observed accelerated population growth, it is forecast that by 2050, approximately 65% of the world’s population will live in urban areas [1]. Growing urban populations result in increasing challenges related to transport, environmental protection, safety, health, education, and public services.

A smart city enables the use of modern technologies to address these challenges, thus contributing to improving the quality of inhabitants’ lives, deepening social integration, and increasing efficiency and environmental responsibility. A smart city enables better use of the existing resources, streamlining the processes occurring in the urban fabric and the adjustment to changing needs and challenges. Thanks to smart technologies, the city is therefore becoming more effective, environmentally friendly, and inhabitant-friendly.

There is not a single, universally accepted definition of a smart city, and since it is a complex concept, it encompasses a variety of social, natural, technical, and humanistic issues. There is a literature review on this topic, which focuses on discussing different approaches to this concept [2,3,4,5,6,7]. Originally, the smart city concept referred to the relationship between the urban space and the use of modern technologies. Currently, the literature mentions the following three most common references to the smart city concept:

- Incorporating information and communications technology (ICT) systems into the urban space;
- Achieving a high quality of life through lasting and sustainable development;
- Smart governance of the city.

A smart city is also differently referred to as an intelligent, informational, or sustainable city or defined as a city of the future or a dream city to live in (Table 1).

Here, is another definition, this time one created by Artificial Intelligence (ChatGPT by OpenAI (San Francisco, CA, USA), an Artificial Intelligence model that enters into dialogue with real users https://chat.openai.com/chat (accessed on 17 January 2023)): its response given to the question “please, write what your opinion is on a smart city” was as follows: “Smart city is a concept involving the use of modern technologies such as the Internet of Things (IoT) networks, big data, smart transport systems, automation, and others, to improve the quality of city dwellers’ lives, increase efficiency and energy savings, and enhance safety. At the same time, it is important, when implementing a smart city project, to take into account aspects of privacy protection, data security, sustainability, and consideration of the needs of different social groups”.

The notion of a smart city is also evolving, with the following successive smart city generations to be distinguished: Smart Cities 1.0, inspired by the available technologies; Smart Cities 2.0, with the decisive role of public administration; Smart Cities 3.0, based on the inhabitants’ creative involvement; 4.0, linked to the concept of Industry 4.0; and even 5.0, linked to the new symbolic notion of Industry 5.0 [14,15,16,17,18,19]. Currently, smart cities are not limited to information and communication technologies, and the centre of gravity has shifted to “smart people” and the corresponding creativity. From this point of view, smart cities focus on improving urban life in the six following dimensions: people, government, economy, mobility, environment and life, and activities of citizens making informed and independent decisions [20]. According to the European Smart City Model, the characteristics of such areas include the following:

- With regard to the economy: entrepreneurship, creativity, innovation, flexible labour market, productivity, international relevance;
- With regard to transport and communication: smart and environmentally friendly transport systems, sustainable transport systems, availability of IT infrastructure, advanced information technologies;
- With regard to the environment: renewable energy, environmental awareness, air quality, energy saving, low CO_2_ emissions, efficient waste management, sustainable use of resources;
- With regard to people: qualifications, open-mindedness, creativity;
- With regard to the quality of life: friendly and clean environment, access to cultural centres, high level of safety, educational opportunities, living conditions, efficient social and technical infrastructure, health care, social welfare;
- With regard to smart governance: public participation, public consultation, participation in public life, transparency of activities, and social and public services.

Therefore, a smart city is a result of the activities of city dwellers, authorities, entrepreneurs, and institutions. Striving to improve the quality of life is supported by both technology and direct activity, as well as the participation of citizens, as social capital is the foundation of development.

This is a truly interdisciplinary field, with the number of publications in this regard constantly increasing [10]. This is confirmed by the analysis of journals from the Web of Science database for the years 2011–2021, which shows that most attention in these publications is given to the technical aspect of a smart city, with publications in the journals dedicated to computer science, engineering, technology, and telecommunications being predominant (Table 2), as their number started to increase rapidly from 2013 onwards.

In summary, the smart city is a process of continuous changes in which the use of smart technologies within the city area is crucial. However, it is also essential that these technologies cooperate with all aspects of the functioning of a city. A city develops thanks to network links between such components as technology, human resources, infrastructure, environment, or the availability of knowledge. A smart city is a city in which the main role is played not only by ICT, but also by human and social capital as well as wise management of natural resources. Therefore, smart cities are distinguished by innovation and development in terms of the improvement of quality of life, care for the environment, the introduction of transport improvements, and urban management skills. It is important, however, that the implementation process is carried out in partnership with inhabitants and considers their needs and opinions. The search for ideas and new solutions frequently takes the form of a city-wide debate and crowdsourcing, which is an increasingly popular way of making assumptions concerning the “city of the future” (Walkowiak, 2015 [21]).

More and more cities are taking steps towards achieving smart city status, as it brings tangible benefits for both inhabitants and entrepreneurs as well as city authorities (see Section 1.1). The achievement of this status is confirmed by the developed smart city indices and rankings prepared on their basis (see Section 1.2). However, it is the inhabitants’ voice, i.e., their opinions on the solutions introduced and their impact on the functioning of a city and the quality of life in the city, that appears to be the most important in this regard. This is crucial because city dwellers are the main beneficiaries of the activities carried out in cities and, by assumption, these activities are intended to improve the quality of their lives. Therefore, research showing the issue of a smart city from the perspective of inhabitants and city managers is important, and such research is carried out, but most of it concerns large cities [22,23,24,25,26].

Taking into account the wisdom and potential of the local community as well as the fact that the proportion of publications relating to the social aspects of smart cities is rather small (see Table 2), a questionnaire survey was conducted in a medium-sized city. The research hypothesis assumed that in the information society era, smart solutions are widely known and used, and inhabitants are actively involved in measures for the development of the city. The aim of the study was to obtain the opinions of the inhabitants (i.e., the main recipients of smart solutions) on the assessment of the smart solutions introduced and the assessment of the functioning of a city as a smart one. The subject of the study was the city of Suwałki, i.e., an example of one of 46 Polish cities with a population ranging from 50,000 to 100,000 inhabitants. An analysis of objective indices classifies the city as smart, and it is included in European lists of smart cities. It is interesting, however, to see how the inhabitants themselves assess the functioning of the city on different planes. Are the facilities introduced relevant to them? Has the quality of life increased? What additional solutions are they awaiting? In addition, the inhabitants’ involvement in city affairs and the level of use of the existing solutions introduced in the city were assessed. The inhabitants were also given the opportunity to assess their level of satisfaction with the individual aspects of the functioning of the city, indicate the areas that require changes, and determine the significance of the smart solutions introduced into the urban structure. The questionnaire survey results allowed an opinion to be formed as to whether the city of Suwałki can be referred to as a smart city of the future and identified the spheres of the city’s activities that need improvement.

### 1.1. Benefits of Being a Smart City

Smart cities are being developed to make people’s lives easier, more comfortable, and safer so that they live in a clean environment and are provided with all the necessary services. With increasing public awareness, more and more city dwellers pay attention to the quality of the urban environment, improvements of functioning within the city’s structure, the ease of moving around, the means of transport employed, and the friendliness of city authorities. People settle where the level of satisfaction and the quality of life are the highest and the range of services offered in the urban space is the best.

**Figure 1:**
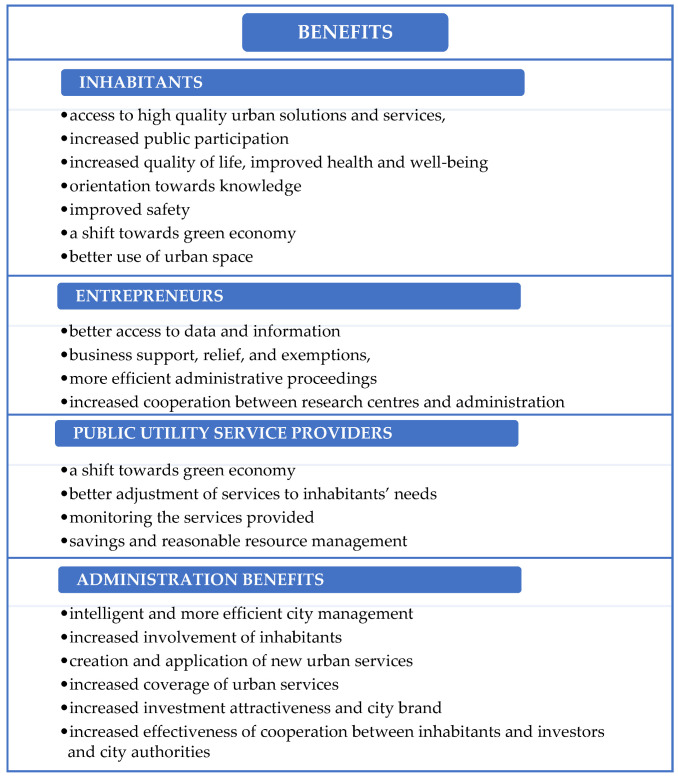
Benefits of having smart city status. Source: own study based on [21,28,29,30,31,32].

The designation of a smart, innovative city also entails an image-related aspect, as it raises the city’s prestige on the international stage and also reflects the satisfaction of inhabitants and entrepreneurs. Innovation opens up many opportunities for development and contributes to increased interest among investors. In this context, seen from the point of view of inhabitants and entrepreneurs, the innovation of a city includes, e.g., the position, modern technology in the infrastructure and transport, tax relief and exemptions, new job opportunities and positions, high wages, courses and training, upskilling, high education and scientific and research facilities, government subsidies and grants, and international cooperation [27]. Figure 1 shows the main benefits of having smart city status according to inhabitants, entrepreneurs, public utility service providers, and city authorities and leaders.

### 1.2. Smart City Indices and Rankings

Every year, a number of rankings are developed, which include cities from all over the world [33]. These are designed using varying criteria and focus on different aspects of the functioning of the city, e.g., some of them assess the quality of life, others the economic level or the technology use level, and still others assess environmental aspects. Some rankings take into account multiple criteria to create universal, standardised lists [34]). An example of such a ranking is standard ISO 37120:2018 Sustainable cities and communities—Indicators for city services and quality of life, prepared by the international organisation World Council on City Data [35]. The standard is aimed at the self-assessment and certification of a city in terms of compliance with the standard and the comparison of cities with each other. However, these indicators are generally adapted to large cities, but indicators for large and medium cities are also developed based on this standard [36]. The main advantage of standard ISO 37120 is not only the measurement of indicators but also the possibility of comparing these results and benchmarking the data between individual cities undergoing this certification [37]. The indicators provided in the standard are used to assess performance and monitor a city’s progress in aspects such as sustainable development and improvement of life quality.

In Poland, the standard was published in 2017 by the Polish Committee for Standardization (PN-ISO 37120 “Zrównoważony rozwój społeczny—Wskaźniki usług miejskich i jakości życia”) [“Sustainable social development—Indicators of urban services and quality of life”]. The Polish Committee for Standardization has also developed the “Programme for the certification of methods for measuring the indicators of urban services and quality of life for the compliance with Polish Standard”. Currently, the ISO 37120 certificate is held by five Polish cities, i.e., Gdynia, Kielce, Warsaw, Lublin, and Gdańsk.

Various rankings of smart cities have been developed. One of them is the IESE Cities in Motion Index, developed by P. Berron and J.E. Ricart from the IESE Business School, University of Navarra, which measures the level of city development in the smart city context. The most recent ranking is from 2022 and concerns 183 cities from 92 countries, of which 85 are capital cities. The ranking was determined based on 114 indicators. The CIMI index is a synthetic indicator based on the weighted aggregation model of the available nine partial indicators, namely Human capital, Social Cohesion, Economy, Governance, Environment, Mobility and transportation, Urban planning, International profile, and Technology. The aim of the CIMI is to help the public and local governments understand the city dimensions and how to manage them [38]. The closer the indicator value approaches 100, the more developed the city is in terms of the smart city concept.

Another example is the European Smart City ranking which takes into account six areas of smart city functioning. The model is under preparation by the Vienna University of Technology (Austria), the Delft University of Technology (the Netherlands), and the University of Ljubljana (Slovenia). The analysis of the smart city level was conducted for the cities that meet the following conditions:

- Average city size (population ranging from 100,000 to 500,000);
- There is at least one higher education institution in the city (an indicator of adequate intellectual capital);
- The city’s impact zone is smaller than 1.5 million inhabitants (this condition is intended to exclude cities dominated by neighbouring large urban agglomerations).

A total of 71 cities were selected for the study conducted in 2007, with the above-mentioned criteria taken into account. The survey analysed 81 indicators making up six areas of the smart city model: smart economy, smart people, smart governance, smart mobility, smart living, and smart environment. The 2013 study also involved 71 cities, while the 2014 study involved 77 cities.

In Poland, the Ranking of Sustainable Polish Cities is under development. The ranking was developed by the company Arcadis in cooperation with researchers from the Wrocław University of Economy and Business and the University of Zielona Góra. The most recent ranking concerns the year 2021. The study assesses the 50 largest cities in Poland in three fundamental areas of sustainable development: society, environment, and economy. The aim of the report is to compare how the designated cities perform in the three specific areas of development. The ranking structure was based on the use of a synthetic indicator whose basis is a set of 45 specific indicators assigned to one of the three dimensions specified above [39].

## 2. Methodology and Area of the Study

The first stage of the study was to analyse the current state of the city of Suwałki. Indicators and statistics acquired from Statistics Poland were analysed [40]. The current status was investigated in areas such as demography, social and economic infrastructure and security, technical infrastructure, and urban space. The analysis of the current and future status of the city, and the smart city level of Suwałki, investigated the Ranking of European Smart Cities, the Ranking of Polish Sustainable Cities Arcadis 2021, local online portals, and the available strategic documents, such as

- A study of determinants and directions of the spatial development of the city of Suwałki [41]—a document specifying the directions of the city’s spatial policy;
- Strategy for Sustainable Development of the City of Suwałki by 2020 and the Suwałki 2030 Development Strategy [42]—documents specifying the vision and strategic goals for the socio-economic development of the city and the main directions of development in line with the current trends resulting from the EU cohesion policy and the decentralisation and globalisation processes occurring in the economy;
- Report on the State of the City of Suwałki 2020 [43]—a document presenting the most important areas of the city’s life: social, economic, and infrastructural, the urban space, and the city management as of the end of 2020;
- Implementation Document for the Strategy for Sustainable Development of the City of Suwałki by 2020 with an outlook until 2023 [44]—a programme document directing the city’s policy in terms of socio-economic development;
- Strategy for Solving Social Problems of the City of Suwałki for the years 2016–2025 [45]—a supplementary document that details the social sphere of the provisions included in the Strategy for Sustainable Development of the City of Suwałki by 2020 [46] and, in particular, the strategic objective 2. Improvement of living conditions.

The second stage was the questionnaire survey conducted among the inhabitants of the city and its immediate vicinity with regard to their perception of the level of smart solutions introduced in the city of Suwałki. To this end, an authors’ original questionnaire was drawn up, targeting the inhabitants of both the city and its immediate surroundings (a neighbourhood within a radius of up to 20 km, functionally linked to the city). A total of 120 respondents took part in the survey. The study was conducted from January 2022 to April 2022 in an online format due to the COVID-19 pandemic. Survey participants were recruited via local web portals and social networks (including the city’s social media channel).

An online anonymous survey questionnaire was drawn up. The questionnaire contained 19 questions, including five questions making up the respondent’s particulars. The questions were open and closed. The questionnaire survey used the semantic differential method, i.e., a quantitative evaluation method developed by C.E. Osgood, G.J. Succi, and P.H. Tannenbaum in 1957 [47]. This is a method commonly applied in the field of sociological, psychological, and geographical sciences, which enables an assessment of a respondent’s impression of, or attitude towards, a phenomenon or an object. The analysis considered pairs of opposing properties defining aspects of the functioning of a city, smart city solutions introduced in the city, and the necessity for change. When compiling the results, each pair of opposing properties was regarded as a continuum comprising five members (from 5—strong positive indication to 1—strong negative indication). The questionnaire was uploaded to the mass media., i.e., to the public Facebook groups “SUWAŁKI—Information” and “Suwałki-ogłoszenia” and to the profile of the information portal “Suwałki.info”, which is among the major information portals of the city of Suwałki.

The analysis of the results takes the form of descriptive statistics. The results obtained in the questionnaire survey were analysed using Statistica software (the results were processed statistically and graphically). The authors’ work included the preparation of figures, graphs, and tables based on the compilation of information derived from analysis of databases, publications, questionnaire surveys, smart city rankings, and the contents of manuscripts dedicated to smart city issues.

## 3. Description of the Subject of the Study

### 3.1. General Characteristics of the City

**Figure 2:**
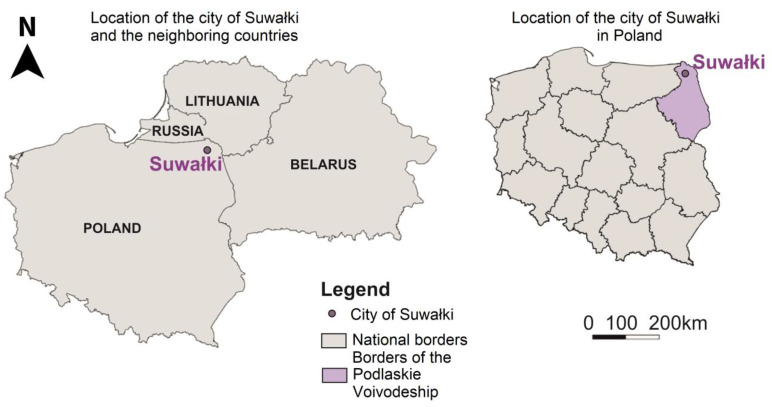
The location of the city of Suwałki compared to neighbouring countries and Poland. Source: own study.

Suwałki is a city with poviat (county) rights, located in north-eastern Poland in the northern part of Podlaskie Voivodeship, as shown in Figure 2. The city is situated close to the border with three countries: Lithuania, Belarus, and Kaliningrad Oblast. According to Statistics Poland data, in 2021, the city with an area of 66 km^2^ was inhabited by 68,839 people, and the population density was 1051 people per km^2^, which makes Suwałki the second largest city in Podlaskie Voivodeship.

In the years 2016–2021, the number of registered unemployed, and thus the registered unemployment rate, gradually decreased, with the exception of 2020. In 2021, the unemployment rate was 5.1%, and the number of registered unemployed amounted to 1437 people. The increase in the number of registered unemployed and the unemployment rate in 2020 was due to the outbreak of the COVID-19 pandemic and the resulting restrictions put in place.

The city of Suwałki and its immediate surroundings are characterised by the occurrence of areas of high natural and landscape values. Due to the location of the city within the “Green Lungs of Poland” area, this region offers great tourist attractions and beautiful landscapes.

Suwałki is an important transport hub, as the city is crossed by transport routes connecting the Baltic countries with Western Europe, the Via Baltica transport route, and the Rail Baltica route. Since 2020, the city has had a ring road and an airport for passenger aircraft with a capacity of up to 50 passengers.

Suwałki has significantly expanded its network of bicycle paths and routes for pedestrians and cyclists. Over the last five years, 34.2 km of bicycle paths have been built. The city is crossed by the Eastern Poland cycling route Green Velo which links five voivodeships. A public bike system, SUWER, was introduced in 2021 to make use of the cycling infrastructure.

### 3.2. Ranking European Smart Cities 3.0 and Ranking of Polish Sustainable Cities Arcadis 2021

**Figure 3:**
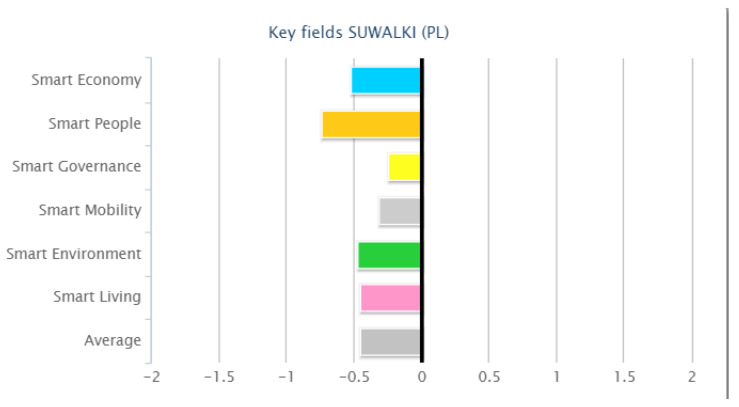
City profile: Suwalki (PL). Source: http://www.smart-cities.eu (accessed on 10 January 2023).

In the European Smart Cities 3.0 ranking of 2014, in the category of medium-sized European cities (population from 100,000 to 500,000 inhabitants), Suwałki was ranked 70th, and scored the following points in the individual categories: Eco: 67, Peo: 70, Gov: 55, Mob: 57, Env: 71, Liv: 68 (Figure 3). The city of Suwałki was an exception to the general rule, as its population was under 100,000 inhabitants.

**Figure 4:**
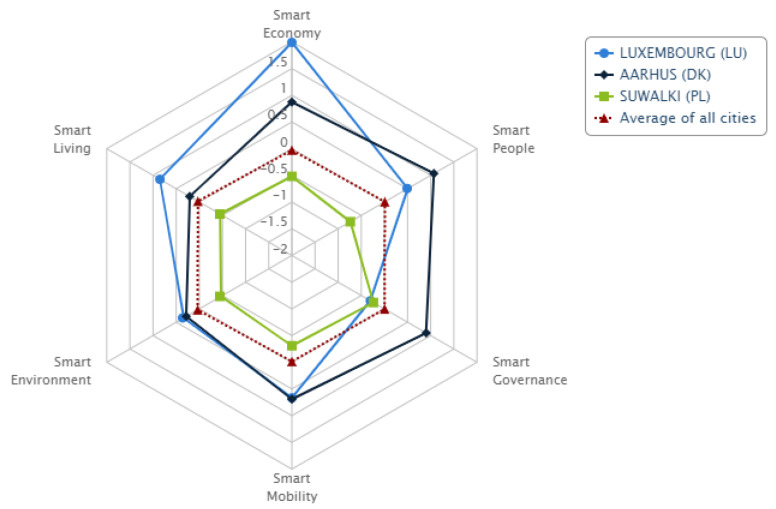
Benchmarking—Luxembourg, Aarhus, Suwałki in six smart city dimensions. Source: http://www.smart-cities.eu (accessed on 10 January 2023).

A total of six Polish cities were included in this ranking. Apart from Suwałki, these are Rzeszów (position 55), Szczecin (position 56), Bydgoszcz (position 62), Białystok (position 66), and Kielce (position 68). The European Smart Cities website provides a preview of benchmarking, i.e., a comparative analysis for three cities in the form of a radar chart. Figure 4 shows a comparison of the cities that took two first positions in the ranking and the Polish city of Suwałki.

**Figure 5:**
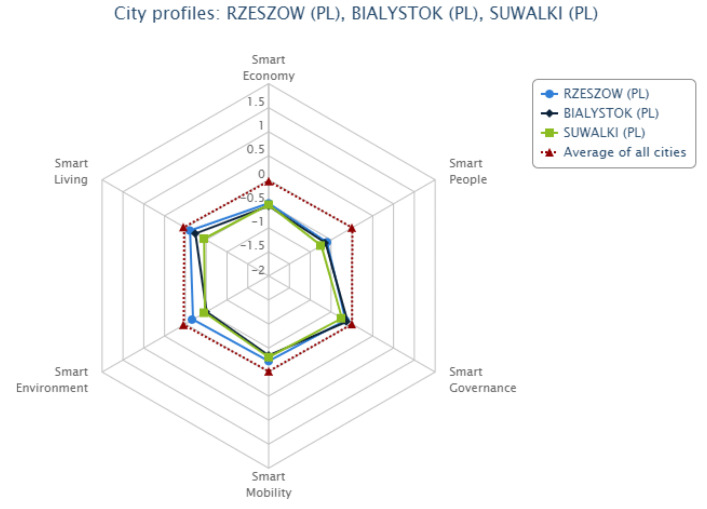
Benchmarking—Luxembourg, Aarhus, Suwałki, Rzeszów, Białystok, Suwałki in six smart city dimensions. Source: http://www.smart-cities.eu (accessed on 10 January 2023).

Figure 5 shows a comparison of three Polish cities, i.e., Rzeszów, Białystok, and Suwałki, due to the fact that these are located in Eastern Poland and covered by the Operational Programme—Development of Eastern Poland (a programme co-funded by EU funds). Based on the 2002 Eurostat study, the regions in which the cities being compared are located were found to have the lowest GDP per capita levels in the European Union.

The 4.0 version of the European Smart City ranking has been available since 2015. Since the city size condition has been changed in this model, cities with population numbers ranging from 300,000 to 1,000,000 could participate, which resulted in the city of Suwałki not being included in the ranking.

In the Ranking of Polish Sustainable Cities Arcadis 2021, Suwałki moved up 11 positions as compared to 2018, from position 25 to 14 (Table 3). This is indicative of the city’s shift towards being smart.

## 4. Study Results

### 4.1. Analysis of the Smart Solutions Implemented in the Urban Fabric

Despite its small size, the city of Suwałki is taking smart measures and introducing solutions in line with the smart city concept into the urban fabric. The city has provided a list of all solutions available to its inhabitants on its website https://um.suwalki.pl (website run by the City Hall in Suwałki; accessed on 15 January 2023), under the tab “For Inhabitants”. Additionally, the website provides a range of important information for entrepreneurs and tourists. The website is a comprehensive source of information and features many links to other portals and digital offices. Access to information about the city is at a good level. The information can be found, e.g., on the Suwałki Public Information Bulletin website (an official City Hall website), in planning and strategic documents and in the Suwałki GeoPortal.

Inhabitants can use the Digital Office, after which they are redirected, without queueing, to the website (https://bezkolejki.eu/umsuwalki, accessed on 15 January 2023), on which they can sign up for the queue system of the Department of Transport and the Registry Office. Using the website https://skm.suwalki.eu offers an opportunity to apply for a Suwałki Inhabitant’s Card, which is a comprehensive programme offering many discounts and benefits, e.g., cheaper tickets to sports facilities, free rides on public transport, discounts for tickets to cultural centres, free kindergartens run by the city, and discounts at the city nursery.

Holders of trusted profiles or qualified electronic signatures can access a variety of electronic government services through platforms such as ePUAP. The Digital Office of the Podlasie Gateway provides information on these services on its website (https://cu.wrotapodlasia.pl, accessed on 15 January 2023). Additionally, municipal authorities also issue electronic ID cards.

Official websites also provide information on current investment conditions. For example, the City Hall website presents information on the current forms of support and relief for entrepreneurs. The city’s assets include the Science and Technology Park and the Suwałki Special Economic Zone.

Moreover, many smart transport and communications solutions have been introduced within the city:

- A modernised parking system offering the possibility of paying by card at parking meters, an option of purchasing an electronic parking ticket via the user’s bank;
- An option of purchasing a public transport ticket using a smartphone, a bank application, or websites designed to find transport connections and plan a journey;
- The “myBus online” application (which tracks public transport vehicles via GPS);
- Electronic information boards located at bus stops, displaying the bus arrival time and its route;
- e-podróżnik and BlaBlaCar systems to facilitate transport;
- The “all red” traffic light system—a smart traffic light system solution including sensors recording vehicle traffic;
- The “green wave” system located in designated streets, which enables a smooth passage provided the car does not exceed the speed of 60 km/h;
- Reconstruction of the airport.

Effective activities of municipal services are also carried out within the city, as patrols are actively carried out both in the daytime and at night. Moreover, the following programs have been developed: the Municipal Crime Prevention Programme and the Programme for the Protection of Citizens and Public Order. The city is also equipped with an urban monitoring system. During mass events, security checks are also carried out using a drone. The city has a modern, environmentally friendly lighting system equipped with traffic sensors. Inhabitants can use web cameras which show the current situation at selected locations in the city.

Smart solutions have also been introduced in the healthcare sector. An option to use tablets and the cloud was introduced at the Suwałki Regional Hospital as early as 2014. The device allows the doctor to have immediate access to complete and comprehensive medical records, assists in emergency situations, and enhances the protection of patient information (https://news.microsoft.com/pl-pl/2014/12/16/w-wojewodzkim-szpitalu-w-suwalkach-lecza-uzywajac-tabletow-i-chmury (accessed on 15 January 2023)).

Suwałki is part of the area known as the Green Lungs of Poland. Due to its location in a naturally and culturally attractive area, Suwałki is a tourism hub. The city provides access to attractive places by means of public transport and increases the length and density of bicycle paths every year. The use of the cycling infrastructure potential has contributed to the introduction of the public bike system SUWER. Bikes can be hired and returned at 16 stations designated throughout the city, and users can choose from 160 bicycles: traditional, tandem, and electric ones (https://um.suwalki.pl/mieszkaniec/komunikacja,3549/suwer-system-roweru-miejskiego,2583990 (accessed on 15 January 2023)). In 2022, electric scooters managed by private companies also appeared on the streets of Suwałki.

In 2021, the city of Suwałki joined the InPost Green City programme, which is part of the InPost company’s long-term strategy under which numerous pro-environmental initiatives are carried out. The implemented programme supports the development of the city in line with the smart city concept by offering inhabitants modern and environmentally friendly services involving a network of self-service parcel pick-up stations. As part of the project, InPost will introduce the following solutions (https://um.suwalki.pl/mieszkaniec/aktualnosci,4002/suwalki-w-programie-inpost-green-city,2584587 (accessed on 15 January 2023)):

- Gradual replacement of vans with electric cars;
- Installation of air sensors;
- Planting air-purifying vegetation on the roofs of self-service parcel pick-up stations.
- Designing innovative anti-smog paving blocks.

Additionally, the company identifies the location of self-service parcel pick-up stations within the urban area so that the setting is compatible with the spatial order concept.

As regards the sphere of culture, inhabitants can browse and purchase tickets at the Suwałki Cultural Centre via the official online portal of the Centre. In addition, the Public Library, on their official website, enables individuals to browse the resources and the available offers and download e-books. The City Hall website annually maintains a calendar of events which presents the most important events planned for a particular year. What is more, the online portal Niebywałe Suwałki maintains a calendar of events that informs about all available events offered in Suwałki in a selected period. Getting to know the city of Suwałki better is possible thanks to a multi-platform application, i.e., the Guide to the City.

What is also important in the smart city concept is the active participation of inhabitants in improving the city through cooperation with the administration. Suwałki inhabitants can submit their ideas in the annually organised Suwałki Participatory Budgeting initiative. Thanks to the live broadcast of the City Council sessions, the inhabitants have greater access to information about the city and the decisions taken by the Council.

Every year for the past six years, the city of Suwałki has been organising a competition for the best diploma thesis related to Suwałki, which contributes to the building of the city’s future. The competition places a strong emphasis on innovation. The city also provides opportunities for additional training and broadening one’s knowledge through courses and training organised, e.g., by the FRESK Foundation, the Foundation of Public Initiatives, and the Association “Nasza Suwalszczyzna”.

As part of the development of the smart city concept, a specialised urban SMS notification system has been launched. The inhabitants can sign up for the system on the City Hall website by entering their phone number and indicating the subject groups of interest. System users can receive important information from the following sections: meteorological warnings, official matters, traffic difficulties/emergencies, educational events, cultural events, and sports events.

Suwałki is an investor-friendly city. Networking of partnerships is undoubtedly facilitated by operating business environment institutions. An example of the activities carried out for the development of economic partnerships is Forum Biznesowe Pogranicza (Borderland Business Forum), a congress event organised annually since 2015, which enables sectoral meetings and exchange of experiences in the areas of leading economic sectors. The events are attended by business, public administration, and business environment institution representatives as well as experts and foreign partners. The city also participates in multiple local authority partnership networks, both internally (inter-municipality relations and in the city itself) and externally (transborder and international cooperation).

In summary, the range of solutions implemented in the city is at a satisfactory level. The city authorities introduced smart amenities and solutions into many aspects of the functioning of the city. There are many more smart city systems and solutions implemented, yet a problem that affects not only the city of Suwałki but also other cities is a lack of aggregated information on the solutions introduced. Therefore, a significant proportion of the public are not aware of the existence of the introduced possibilities and amenities.

### 4.2. Questionnaire Survey Results—Analysis of the Smart City Level for Suwałki from a Societal Perspective

The questionnaire survey involved 120 respondents, of whom 75% declared residing within the city of Suwałki, while 25% lived within a radius of up to 20 km of the city limits.

**Figure 6:**
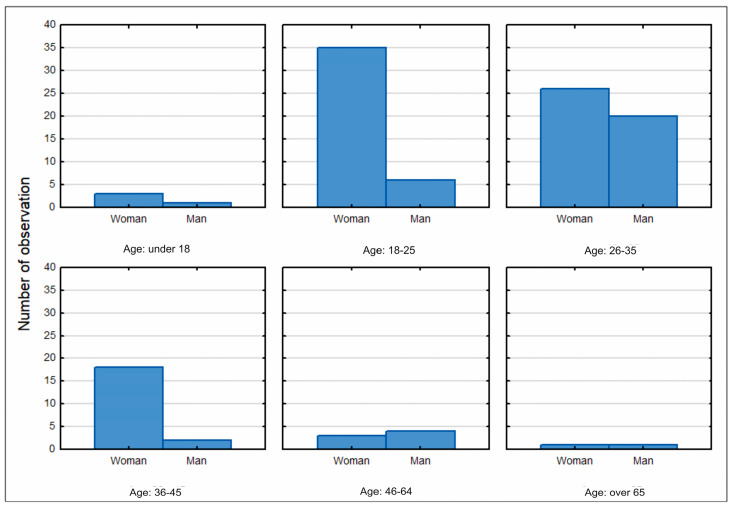
Histogram of the distribution of the survey respondents by gender and age. Source: own study based on questionnaire surveys.

Women (86) accounted for 72% of the respondents, while the proportion of men (34) was 28%. People from the age groups of 26–35 years and 18–25 years represented the greatest proportion of respondents in the survey, with the ratio of women to men in these age brackets being higher (Figure 6). The smallest group taking part in the survey was people aged over 6.

**Figure 7:**
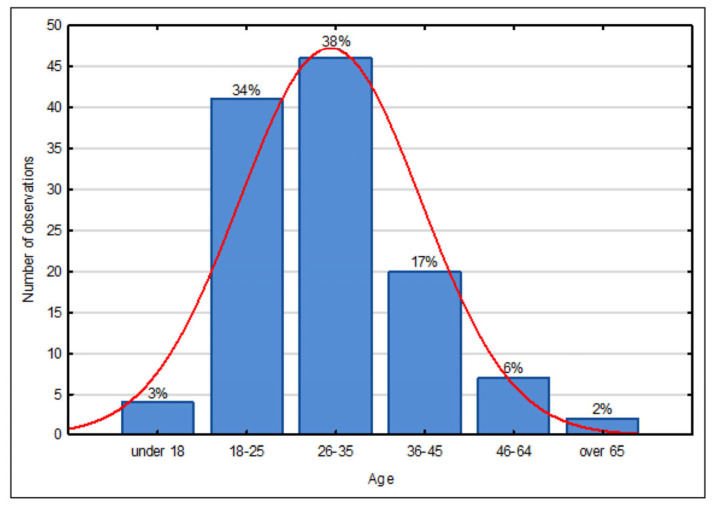
Histogram of the age of respondents. Source: own study based on questionnaire surveys.

The distribution of the age of respondents is similar to a normal distribution, as shown in Figure 7.

**Figure 8:**
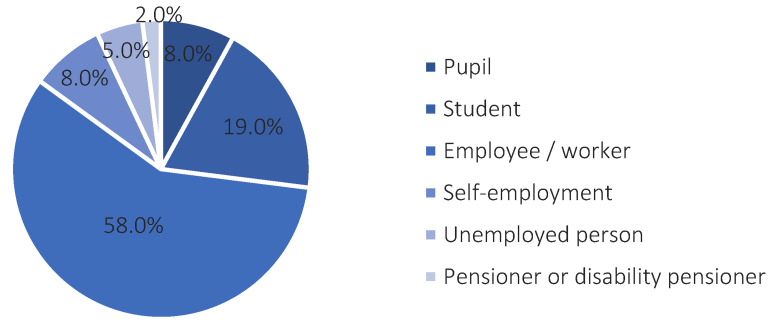
The structure of the professional status of respondents. Source: own study based on questionnaire surveys.

The majority of respondents were people with a tertiary education degree (54.2%), working, in the 26–35 age bracket. The smallest percentage in the group under study were people with primary (4.2%) and vocational education (5.8%). The remaining group of respondents with a secondary education accounted for 35.8%. The structure of the respondents’ professional situation is provided in Figure 8.

An analysis of survey responses indicated that the majority (75%+) of respondents do not participate in city affairs or attempt to influence decision-making. Only less than 20% take part in surveys and studies and are active on online forums. At the same time, the respondents are interested in current information about what is going on in the city. However, most of them are not active in public life (49.2% answers), or they participate only in anonymous studies and surveys and contribute anonymously to online forums (40%). Only 10% are actively involved (petitions, strikes, etc.).

**Figure 9:**
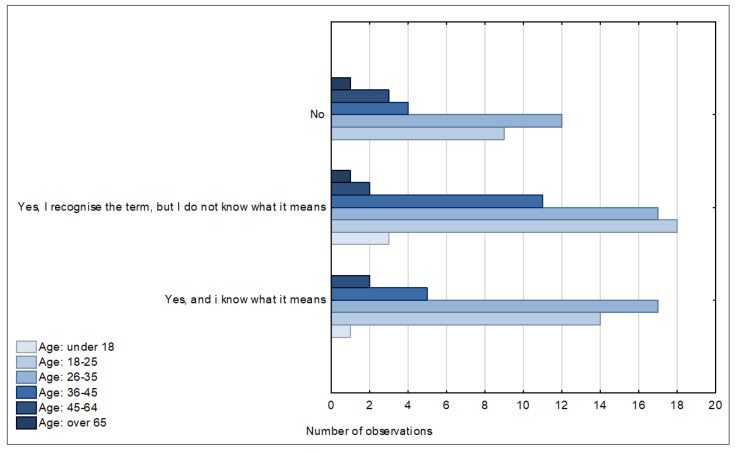
Awareness of the term “smart city” in different age groups. Source: own study based on questionnaire surveys.

The respondents were asked the question: “Have you ever heard the term ‘smart city’?” A total of 25% of the respondents did not know this term, while 43% had heard the term but did not know its meaning, which particularly concerns people from the age group of over 65 years. The largest group of respondents who knew and understood this term were those from the age brackets of 26–35 years (Figure 9).

**Figure 10:**
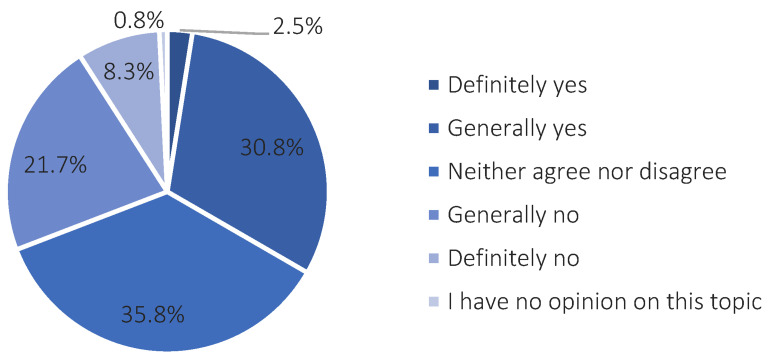
An assessment of the match between the term smart city and the city of Suwałki. Source: own study based on questionnaire surveys.

After providing the respondents with a short general definition of a smart city, which reads as follows, “A smart city is a city that makes investments in human, social, and infrastructural capital. These measures are aimed at ensuring a high quality of life and promoting sustainable development and wise management of natural resources”, they were asked to determine, according to their own assessment, if the city of Suwałki could be described as a smart city. Approximately 33% of the respondents answered “yes” (the answers “definitely yes” and “generally yes”) (Figure 10).

**Figure 11:**
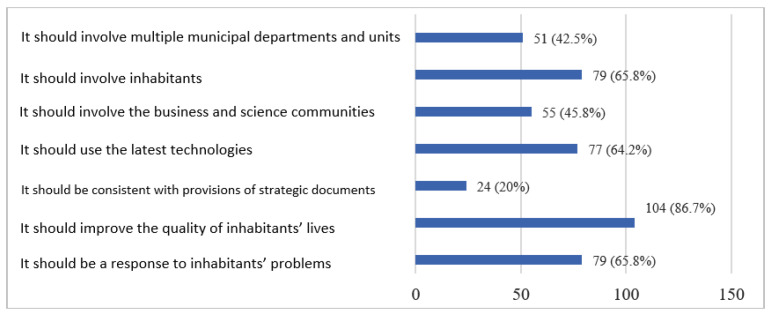
The expected smart city project criteria. Source: own study based on questionnaire surveys.

The introduction of smart city solutions and amenities should be well thought out. The respondents indicated the criteria that a smart city project should meet. According to the respondents, the most important aspect is a positive effect on the quality of life. They also pointed out as crucial that the projects should be a response to the inhabitants’ problems and that it should involve them (Figure 11). Respondents who had the opportunity to provide their own answers pointed out that such a project should take into account the protection of natural resources and nature, which means that taking care for the natural environment is important to inhabitants. They also indicated that numerous investments should be realised, but only those that are needed will bring real effects and have utility values.

The assessment of the smart city level is influenced by the level of satisfaction with the current spatial policy and urban management policy. More than half of the respondents (53.3%) declared that the current development policy was satisfactory, but the city still needed some changes, while 4.2% of the respondents were fully satisfied with the development policy. A medium level of satisfaction was declared by 33.3% of the respondents, while 9.2% stated that the activities carried out in the city were unnecessary and in no way improved the functioning of the city. The overall level of satisfaction with the policy pursued by the city authorities is satisfactory.

The question about the level of satisfaction with individual aspects of the functioning of the city used the itemised scale summary tool of the Statistica program, with the limits set above 30% (gray background color in the cells Table 4 and Table 5). It can be seen from the analysis results that the largest number of respondents are satisfied with the level of access to green areas, access to education, cleanliness of city streets, and the quality of bicycle paths and routes for pedestrians and cyclists. The most common choices among the respondents’ answers analysed included “satisfied” or “neither satisfied nor dissatisfied” (Table 4).

**Figure 12:**
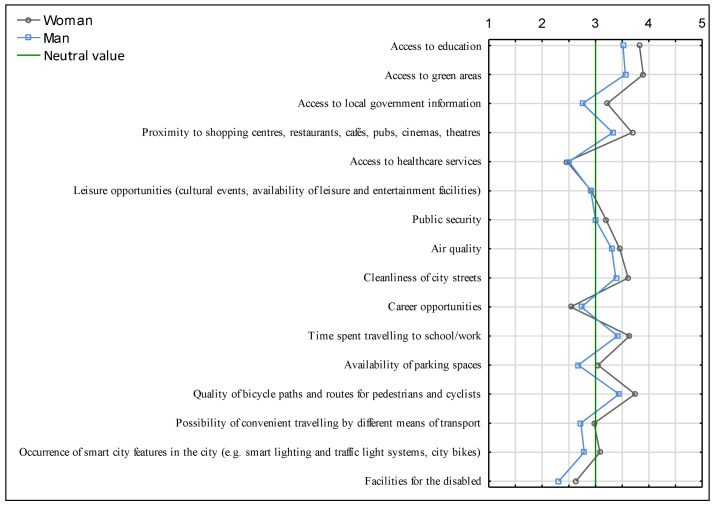
Semantic differential of the level of satisfaction with individual aspects of the functioning of the city of Suwałki. Source: own study based on questionnaire surveys.

A mean value analysis using a semantic differential was used to construct a profile of respondents. According to men, access to local government information, access to healthcare services, leisure opportunities, career opportunities, availability of public spaces, the possibility of convenient travelling by different means of transport, the occurrence of smart city facilities in the city, and facilities for the disabled ranked below the neutral value. According to women, the aspects below the neutral value include access to healthcare services, career opportunities, the possibility of convenient travelling by different means of transport, and facilities for the disabled. Having analysed the semantic graph presented in Figure 12, it can be noted that women assess the urban space of Suwałki better, since the average rating, except for the aspect of access to healthcare services and career opportunities, is higher than that of men.

An analysis of data on the utilization of amenities added to Suwałki’s urban space shows that a significant portion of residents are unaware of these solutions. This is confirmed by the fact that in the previous question, the aspect concerning access to local government information was assessed below the neutral value. Moreover, some amenities are known to the inhabitants, yet they are not used, which may be due to poor promotion and the low attractiveness of these facilities. E-payments are the most widely utilized solution introduced in the city (Table 5).

**Figure 13:**
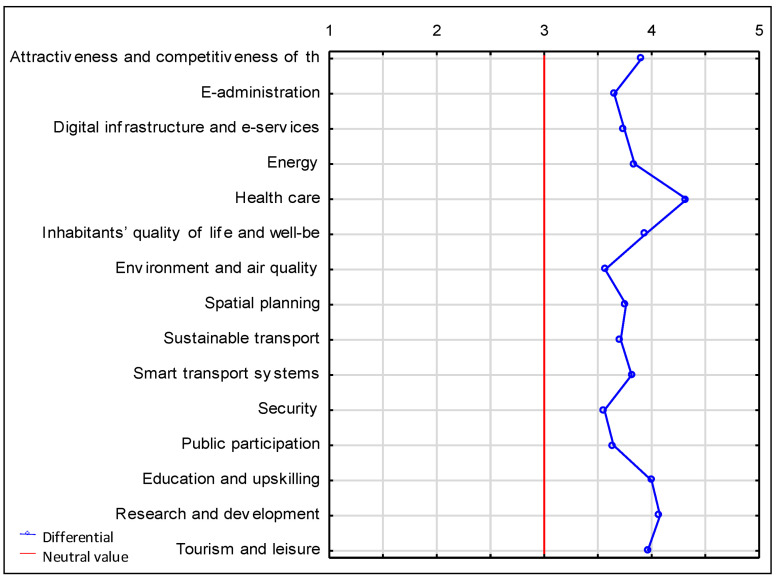
Semantic differential representing the necessity of introducing changes in individual aspects of the functioning of the city of Suwałki. Source: own study based on questionnaire surveys.

An analysis of the semantic differential reveals that residents of Suwałki believe changes should be implemented in all aspects. However, by far the largest number of people indicated that changes were particularly necessary in the healthcare sector, with a mean rating of 4.33 (on a scale of 1 to 5). The inhabitants noted the least need for a change in public security (Figure 13).

**Figure 14:**
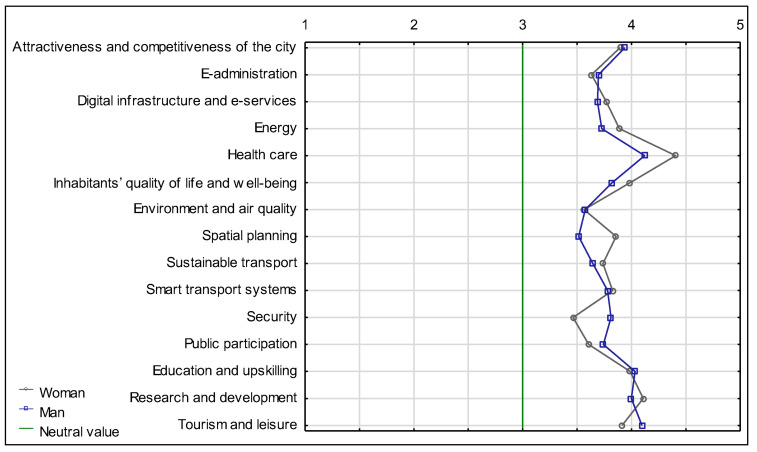
Semantic differential for the necessity of introducing changes in individual aspects of the functioning of the city of Suwałki, by gender. Source: own study based on questionnaire surveys.

The semantic differential graph shows that the highest demand for change comes from respondents aged 46–64, while women are the dominant gender group (Figure 14).

**Figure 15:**
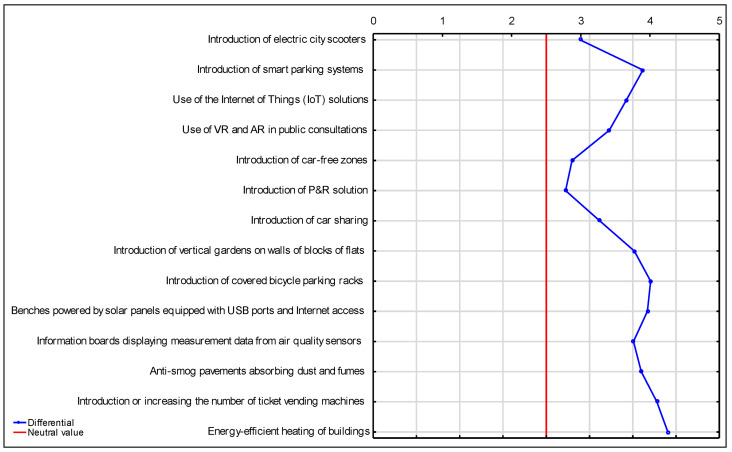
Semantic differential for the assessment of the relevance of introducing smart solutions in the city of Suwałki. Source: own study based on questionnaire surveys.

According to the respondents, the solutions recommended for implementation in the city include energy-efficient heating of buildings, the introduction of ticket vending machines in public transport or increasing the number of ticket vending machines at bus stops, and the introduction of covered, rain-protected bicycle parking places/racks. Of all the proposed solutions, the P&R solutions appear to be the least worthwhile, which may be due to the city’s area being too small. This, however, may change in the future due to the expansion of urban areas (Figure 15).

**Figure 16:**
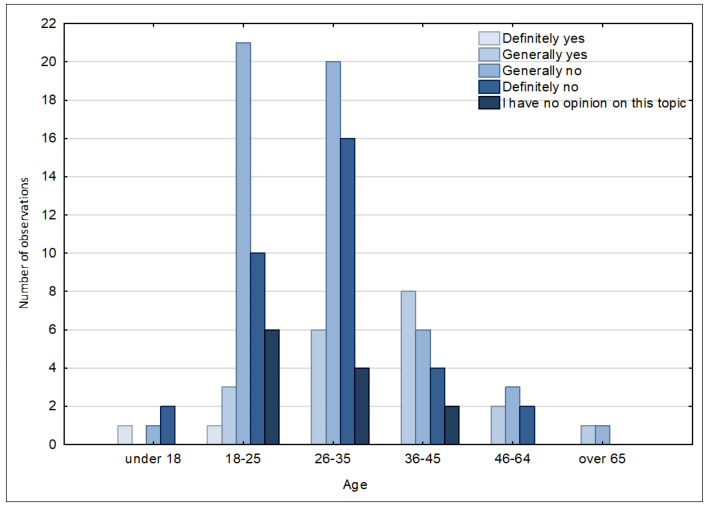
Assessment of the level of awareness of the smart city measures being taken by the city of Suwałki by age category. Source: own study based on questionnaire surveys.

Respondents were also asked about the level of their awareness of the smart city measures being taken by the city authorities. The majority of respondents stated that the level was not sufficient. The answers “generally no” and “definitely no” were provided by 43.3% and 28.3% of the respondents, respectively. The answers “definitely yes” and “generally yes” were provided by 1.7% and 16.7% of the respondents, while the others (10%) had no opinion on the topic. This fact concerns the answers given to the question, “Please indicate which of the smart city solutions mentioned below you have noticed in the city of Suwałki”; many inhabitants were not aware of the existing solutions. Additionally, having analysed the answers in particular age groups, it can be noted that people aged 18–35 years believe that the city fails to inform significantly, as the information opportunities are not used, and the outreach to all age groups is not at a satisfactory level (Figure 16).

The vast majority of respondents (93.4%) answered “no” to the question “Are you concerned about the Smart City concept to be implemented in the city?”, of whom 59.2% were definitely not concerned about such solutions. Only 5% of respondents have concerns in this regard, while the others have no opinion. Therefore, inhabitants are very receptive to such solutions.

**Figure 17:**
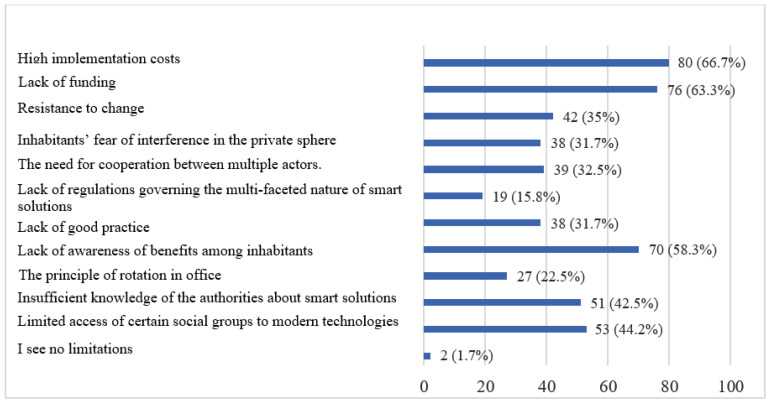
Obstacles encountered in implementing a smart city project according to respondents. Source: own study based on questionnaire survey.

Since the smart city concept has certain limitations in its implementation, the respondents were asked to identify the barriers they believed were major. The respondents indicated high implementation costs and lack of funding as the main barriers. They also considered that the inhabitants are not fully aware of the benefits brought by the introduced solutions, which makes the interest in the smart city concept low, and thus the authorities receive no information on the need to improve the city space (Figure 17).

In order to identify the direction of development and a model city, the inhabitants were asked which Polish city they liked the most. The cities most frequently indicated by the respondents included Gdańsk (21 indications), Suwałki (20 indications), Wrocław (19 indications), and Kraków (17 indications). Such a large number of indications for Suwałki confirms that, according to the respondents, it is a city offering good living conditions.

## 5. Summary and Conclusions

The ever-increasing interest in the city as a place to live, and consequently its continued growth and the expansion of its boundaries, results in a growing interest in the smart city concept that is intended to respond to the challenges of improving the quality of life in a modern city. Smart measures are aimed at meeting the challenges of civilisation progress through achieving the smart city status. The solutions being introduced into the urban structure should provide a response to inhabitants’ problems and improve their comfort and quality of life. In smart cities, ICT plays the main role, but human and social capital, as well as wise management of natural resources, have an enormous effect as well. Defining the problems of a city and a smart city and learning about the possibilities and limitations of smart cities enabled the assessment of the city of Suwałki in terms of the level of implementation of the solutions in this regard.

The study allowed the inhabitants’ expectations, problems, and needs to be identified. The survey questionnaire allowed the inhabitants to express their opinions on their perception of the city and to assess the level of development in recent years as well as the smart city solutions introduced into the urban fabric. Moreover, the study results also identified the areas which, in the inhabitants’ opinion, require changes and indicated the priority areas in which measures should be implemented.

When considering the three perspectives of city analysis in the smart city context, the following can be concluded about the city of Suwałki:

- From the perspective of definition and literature—the city meets the determinants resulting from the definition of this term, which is confirmed by its inclusion in the Ranking of European Smart Cities 3.0 and in the Ranking of Polish Sustainable Cities Arcadis 2021 (in which it takes an increasingly higher position).
- From the perspective of an analysis of a small city in terms of smart solution functioning—the analysis of strategic documents and the analysis of smart solutions implemented in the urban fabric also confirm that the range of solutions implemented in the city is at a satisfactory level.
- From the inhabitants’ perspective—inhabitants are satisfied with the functioning of most smart solutions in the urban space and have a positive attitude towards the implementation of the smart city concept while indicating the insufficient level of informing about these solutions.

Based on the survey, it can be concluded that the city has introduced numerous amenities to facilitate and improve the quality of life. Inhabitants are satisfied with the functioning of most aspects of the urban space, but they are also noticing problems that require intervention. The priority action to be taken by urban communities should be to improve health care and development opportunities. Further, the most desirable solutions following health care include energy-efficient heating of buildings, ticket vending machines in public transport, and covered rain-protected bicycle parking places/racks. On the other hand, the least popular solution is park and ride. Knowing the public’s position, city authorities may focus on the introduction of changes in those areas where they are most desired by inhabitants.

It is also important to encourage inhabitants to be involved in public participation initiatives, as this will have a positive impact on urban space management and, thus, on the level of city dwellers’ satisfaction. Failure to participate and be involved in local matters results in a feeling of separateness and belonging to no community and a low level of shared responsibility for the city’s affairs. Participation is the informed involvement of citizens in matters that are important to them, through which they gain influence (and some degree of control) over the authorities’ actions. As regards the criteria that a smart city project should meet, among the most frequent responses were those stating that the effect of its implementation should be improved quality of life and that the projects should respond to inhabitants’ needs. City administrators should also consider an effective solution for informing inhabitants about the smart solutions and services in place in the city. The awareness of the local community about these solutions is low, but the goal is to increase their utilization. Inhabitants are not always fully aware of the benefits brought by the solutions introduced, which decreases interest in the smart city concept, and thus the authorities receive no feedback on the need for further solutions to improve the city space. The city authorities should therefore focus on better informing inhabitants of their activities using a variety of communication channels to reach all inhabitants.

The questionnaire survey results and analysis show that Suwałki has many amenities that enhance the functioning of its residents, tourists, and businesses. The level of inhabitants’ satisfaction with individual aspects of the functioning of Suwałki’s urban space is high. The local community notes deficiencies in a few areas, which should not be underestimated. City managers should learn about inhabitants’ problems and respond to their needs and expectations. Improving the functioning of the city, implementing additional amenities, and making full use of all the advantages and potential will help Suwałki develop, increase its prestige, and allow it to be referred to as a smart city.

In summary, measures taken in the city are important and bring positive results. However, this is not sufficient for Suwałki to be called a fully smart city. It can be concluded that the city has significant potential for improvement, as the majority of the respondents are not afraid of the implementation of the smart city concept. The Suwałki inhabitants are open to the introduction of the Smart City concept, but they need more precise information on the measures taken and the benefits they will bring. This is important because an increase in the level of quality of life in the city in line with the concepts of smart cities and sustainable development affects the quality of inhabitants’ lives. A certain correlation occurs in cities: the higher the city’s smartness level, the more rapidly its population increases. A friendly and easy city lifestyle attracts a larger portion of the population to live and function in the city. These are extremely important aspects, particularly for medium-sized cities, which generally face the problem of depopulation. 

Having analysed the collected information and trends concerning the city of Suwałki, the following priority measures in several areas can be identified:

- The need to invest in health care as a priority area requiring change;
- The need to improve informing inhabitants about Smart City measures and their benefits; to this end, a variety of communication channels need to be used, including websites, social networks, leaflets, etc.;
- The need to increase the number of ticket vending machines in public transport and to introduce covered, rain-protected bicycle parking places/racks to facilitate the use of public transport and bicycles;
- The need to invest in smart energy systems and building heating systems, which will contribute to environmental protection and energy savings, and will improve the inhabitants’ life quality;
- The development of the inhabitants’ digital skills, as in the era of accelerating technological development it is important for them to increase their knowledge and skills in modern technologies, which could include learning how to program, design websites, manage data, or use e-commerce platforms;
- The development of civic attitudes and increasing the inhabitants’ involvement in city affairs.

Similar questionnaire surveys concerning smart solutions were also conducted among inhabitants in other Polish cities: Zabrze [48], Poznań [49], Gliwice and Zabrze [50], and the cities and municipalities being part of the Metropolis GZM [51], Kraków [52]. These also indicate the increasing implementation of smart solutions and their contribution to the improvement of life quality. The conclusions of social and sociological research into the smart city concept are that the social dimension should be an integral part of smart city projects. This means that when designing and implementing smart city technologies, the inhabitants’ perspective and needs must be taken into account. Sociological research can help to understand how smart cities affect the quality of life and the inhabitants’ involvement in the decision-making process and what opportunities inhabitants have to express their opinions and suggestions regarding smart city projects.

In conclusion, it should be emphasised that solutions leading to the development of numerous plans which improve the quality of life, reduce administrative costs, and increase the efficiency of infrastructure, leading to civilisation progress, allow a city to be referred to as a smart city, yet the achievement of this status is not guaranteed. Due to the continuous development of modern technologies and the emergence of new solutions and opportunities, the smart city is a concept that is continuously being pursued according to the Deming cycle: plan, do, check, act [53]. These technologies include modern 3D and virtual twin solutions [54,55,56,57,58,59,60]. By combining the real and virtual worlds, these technologies support the development and management of the urban structure and improve the quality of public consultation. These innovative solutions are finding their use in cities of the future as they contribute to an improvement in the functioning, safety, and management of a city. Due to the increasing complexity and pace of urban development, it is becoming important to be able to predict spatial impacts at the investment planning stage, which is made possible thanks to the use of these technologies in urban management.

## Figures and Tables

**Table 1:** Example definitions of modern city concepts.

Name	Definition	Authors
smart city	“Smart cities represent a conceptual urban development model based on the utilization of human, collective and technological capital for the enhancement of development and prosperity in urban agglomerations.”, p. 1.	Angelidou, M. [8]
digital city	“Digital cities integrate urban information (both achievable and real-time) and create public spaces for people living in the cities”, p. 1	Ishida, T. [9]
intelligent city	“On the ground of innovative agglomerations and digital spatialities, the Intelligent City is becoming the dominant urban development and planning paradigm of the twenty-first century, connecting urban, innovation, and digital environments and setting in motion powerful socio-technological engines for change and sustainable growth”, p. 2 “Intelligent cities constitute a discrete category of intelligent environments generated by the agglomeration of creativities, smaller systems of innovation that operate within cities (technology districts, technology parks, innovation poles, innovative clusters), and digital networks and online services. Their added value lies in their ability to bring together three forms of intelligence: the human intelligence of the city’s population, the collective intelligence of innovation support institutions, and the artificial intelligence of digital networks and online services”, p. 19	Komninos, N. [10] Komninos, N. [11]
knowledge city (informational cities)	“A knowledge city is a city that aims at a knowledge-based development, by continuously encouraging the KM processes. This can be achieved through the continuous interaction between its knowledge agents themselves and at the same time between them and other cities’ knowledge agents. The city’s appropriate design, ICT networks and infrastructures support these interactions”	Ergazakis, K., Metaxiotis, K. & Psarras, J. [12]
sustainable cities	“In summary, sustainable cities are cities where socio-economic interests are brought together in harmony (co-evolution) with environmental and energy concerns in order to ensure continuity in change”, p. 4.	Nijkamp, P., Perrels, A. [13]

**Table 2:** Ranking of journals from the Web of Science database in the years 2011–2021 according to the key for the occurrence of the smart city concept.

Year				2011	2012	2013	2014	2015	2016	2017	2018	2019	2020	2021
Journal Name/Total Number of Publications				549	879	1943	2181	2570	4521	5777	7745	8283	6714	7371
Engineering Electrical Electronic														
Computer Science Theory Methods														
Computer Science Information Systems														
Telecommunications														
Computer Science Artificial Intelligence														
Computer Science Interdisciplinary Applications														
Green Sustainable Science Technology														
Urban Studies														
Energy Fuels														
Computer Science Hardware Architecture														
Instruments Instrumentation														
Automation Control Systems														
Environmental Sciences														
Environmental Studies														
Materials Science Multidisciplinary														
Transportation Science Technology														
Engineering Civil														
Computer Science Software Engineering														
Regional Urban Planning														
Engineering Multidisciplinary														
Economics														
Geography														
Management														
Architecture														
Business														
Construction Building Technology														
Engineering Environmental														
Operations Research Management Science														
Social Sciences Interdisciplinary														
Transportation														
where														
									
no ranking	<5	[5–10]	[10–15]	[15–20]		[20–25]		[25–30]		[30–35]		[35–40]		>40

**Table 3:** Overall classification broken down by development areas of the Ranking of Polish Sustainable Cities Arcadis 2021.

Position	City	Result [%]	Society [%]	Position	Economy [%]	Position	Environment [%]	Position
1	Warszawa (capital city)	68.72	26.03	1	16.83	42	25.87	1
2	Toruń	67.22	22.94	12	23.17	11	21.11	7
3	Zielona Góra	66.17	23.89	9	24.92	4	17.36	24
4	Rzeszów	65.95	24.29	4	20.95	21	20.71	8
5	Poznań	65.81	24.60	3	18.10	36	23.11	4
6	Bielsko-Biała	65.49	21.43	15	24.29	5	19.78	10
7	Olsztyn	65.20	23.57	10	23.65	9	17.98	20
8	Gdańsk	65.14	20.95	17	20.48	24	23.71	2
9	Koszalin	64.97	23.33	11	25.40	1	16.24	31
10	Lublin	64.40	24.29	5	21.43	20	18.69	13
11	Białystok	64.36	23.89	8	22.38	14	18.09	18
…	…………	…	…	…	…	…	…	…
14	Suwałki	61.96	20.08	21	23.97	7	17.91	21
…	…………	…	…	…	…	…	…	…

Source: own study based on [39].

**Table 4:** The level of inhabitants’ satisfaction with individual functional aspects of the city of Suwałki.

To What Extent Are You Satisfied with the Following Functional Aspects of the City of Suwałki?	Very Dissatisfied	Dissatisfied	Neither Satisfied nor Dissatisfied	Satisfied	Very Satisfied	I Have no Opinion on This Topic
Access to education	1.7%	11.7%	23.3%	35.8%	25.8%	1.7%
Access to green areas	3.3%	11.7%	20.0%	31.7%	33.3%	0.0%
Access to local government information	5.8%	20.8%	36.7%	24.2%	8.3%	4.2%
Proximity to shopping centres, restaurants, cafés, pubs, cinemas, theatres	5.8%	11.7%	28.3%	26.7%	27.5%	0.0%
Access to healthcare services	17.5%	38.3%	30.0%	7.5%	6.7%	0.0%
Leisure opportunities (cultural events, availability of leisure and entertainment facilities)	12.5%	28.3%	26.7%	20.0%	12.5%	0.0%
Public security	10.0%	22.5%	24.2%	28.3%	14.2%	0.8%
Air quality	8.3%	15.8%	21.7%	33.3%	20.0%	0.8%
Cleanliness of city streets	5.0%	14.2%	22.5%	36.7%	20.8%	0.8%
Career opportunities	23.3%	25.0%	29.2%	14.2%	8.3%	0.0%
Time spent travelling to school/work	5.8%	13.3%	21.7%	32.5%	24.2%	2.5%
Availability of parking spaces	20.0%	14.2%	22.5%	33.3%	7.5%	2.5%
Quality of bicycle paths and routes for pedestrians and cyclists	5.0%	10.8%	20.8%	35.0%	24.2%	4.2%
Possibility of convenient travelling by different means of transport	13.3%	22.5%	31.7%	18.3%	10.8%	3.3%
Occurrence of smart city features in the city (e.g., smart lighting and traffic light systems, city bikes)	10.0%	16.7%	40.8%	23.3%	6.7%	2.5%
Facilities for the disabled	15.8%	29.2%	25.8%	12.5%	4.2%	12.5%

Source: own study based on questionnaire surveys.

**Table 5:** Awareness of smart city solutions and the level of their use in the city of Suwałki.

Amenities	I Have Noticed Them but Do Not Use Them	I Use Them Occasionally	I Use Them Frequently	I Have Not Noticed Them
Access to free wireless Internet	48.3%	15.0%	2.5%	34.2%
Possibility of buying an electronic bus ticket	57.5%	14.2%	3.3%	25.0%
Electronic notice boards	34.2%	25.0%	15.8%	25.0%
Checking and tracking public transport via an application	35.0%	12.5%	9.2%	43.3%
Modernised parking system (payment by card, moBiLET electronic ticket)	27.5%	19.2%	10.8%	42.5%
E-administration	33.3%	24.2%	18.3%	24.2%
E-payments	23.3%	32.5%	30.0%	14.2%
City bikes	50.0%	34.2%	13.3%	2.5%
Urban SMS Notification System	30.0%	15.0%	8.3%	46.7%
Traffic amenities (“all red” light system and a green wave)	12.5%	17.5%	20.0%	50.0%
Smart City Lighting	16.7%	14.2%	12.5%	56.7%
Urban monitoring, web camera	39.2%	16.7%	3.3%	40.8%

Source: own study based on questionnaire surveys.

## Data Availability

Not applicable.
